# Sustained degradation of quality of life in a subgroup of lymphoma survivors: a two-year prospective survey

**DOI:** 10.1186/s12885-019-6337-2

**Published:** 2019-12-03

**Authors:** Gisèle Compaci, Cécile Conte, Lucie Oberic, Loïc Ysebaert, Guy Laurent, Fabien Despas

**Affiliations:** 1 Department of Hematology - Internal Medicine, Toulouse University Hospital, Cancer University Institute of Toulouse Oncopôle, Toulouse, France; 2 Service of Medical and Clinical Pharmacology, Center of Pharmacovigilance, Pharmaco-epidemiology and Information on Drugs, Toulouse University Hospital, 37 Allées Jules Guesde, 31000 Toulouse, France; 3 Laboratory of Medical and Clinical Pharmacology Faculty of Medicine, University III Paul Sabatier, Toulouse, France; 4 INSERM Unit 1027, Faculty of Medicine, The French National Institute of Health and Medical Research, Toulouse, France; 5 INSERM Unit 1037, Center of Cancer Research, The French National Institute of Health and Medical Research, Toulouse, France

**Keywords:** Cancer survivorship, Lymphoma, Anthracycline-based chemotherapy, Shared care model, Quality of life

## Abstract

**Background:**

Previous studies have suggested that lymphoma survivors commonly display altered Health-Related Quality of Life (HRQoL). Because these were predominantly cross-sectional studies, the dynamic of events as well as the factors which influence HRQoL remain to be determined.

**Methods:**

We conducted a prospective study on a cohort of 204 Hodgkin and non-Hodgkin lymphoma survivors who remained disease-free 2 years after undergoing chemotherapy (referred to the M0-M12-M24 periods).

**Results:**

We found that although Physical and Mental Component Scores (PCS and MCS) of HRQoL significantly improved from M0 to M24 in the vast majority of patients (favorable group), approximately 20% of patients displayed severe alterations in HRQoL (global SF-36 scores < 50) extending over the 2-year period (unfavorable group). Low M24 PCSs were associated with Post-Traumatic Stress Disorder (PTSD), depression, cardiovascular events and neuropathy. In contrast social determinants, comorbidity and infections, as well as several other parameters related to the disease or to the treatment itself were not associated with low M24 PCSs. Low M24 MCSs were associated with a low educational level, aggressive histology, infections, cardiovascular events and PTSS. However, the most predictive risk factor for low SF-36 scores at M24 was a low SF-36 score at M12. The unfavorable group also displayed a low incidence of return to work.

**Conclusions:**

Although the HRQoL of lymphoma survivors generally improved over time, persistent and severe HRQoL alterations still affected approximately one fifth of patients, resulting in important social consequences. This specific group, which presents with identifiable risk factors, may benefit from early, targeted psycho-social support.

## Background

Both Non-Hodgkin and Hodgkin Lymphomas (NHL and HL, respectively) are both considered to be very chemo-sensitive cancers. HLs are cured in more than 85% of patients, including advanced forms of the disease, due to the remarkable efficacy of ABVD or/and BEACOPP regimens [1]. For NHL, the standard RCHOP21 or RCHOP14 regimens approximately yield an 80% response rate, with the majority of cases achieving a complete response (CR) [2]. Chemotherapy-related toxicity, during the active phase of treatment has decreased over the last few decades and the current toxicity death rate does not exceed 1–3% [3]. The rate of relapse is below 10% for HL and ranges from 10 to 20% for most NHLs, depending on risk factors and histological subtype, with the exception of more aggressive forms such as T-cell derived NHL (10% of cases) [4]. Although therapy is potentially associated with acute toxicities such as sepsis, mucitis, fatigue and cytopenias, which sometimes require transfusions, chemotherapy toxicity remains generally acceptable as reflected by the high rate of dose adherence [5].

Based on these findings and considerations, it should be possible to predict favorable outcome. However, this expectation has been contravened by a number of studies which have reported that the post-treatment trajectory is frequently disrupted by neuropathy or infections, occurrence of non-hematological diseases including cardiovascular events or second cancers, even during the early stages of survivorship [6]. Moreover, socio-psychological complications, such as chronic fatigue, also occurred notably in HL [7, 8], as well as mental disorders such as anxiety, depression, fear of relapse, and Post-Traumatic Stress Disorder (PTSD) [9] as well as occupational difficulties [10]. All these components impact on health-related quality of life (HRQoL) and slow down the return to the norm for both HL [11] and NHL [12] patients. This may explain why the HRQoL of lymphoma patients is relatively poor, particularly when compared to other cancers that have worse prognoses such as lung cancers, renal cancers [13] and other blood neoplasias [10]. This also may explain why informal caregivers play such an important supportive role [14].

Most of these studies are cross-sectional, with prospective studies much less common. The latter still provide information about the event dynamics of NHL survivorship and determine whether some initial features related to the disease, the treatment or the patient (including social determinants) affect HRQoL along the post-cancer trajectory.

We described the AMA-AC (Ambulatory Medical Assistance for After Cancer) in one of our previous reports [6]. AMA-AC is derived from the patient navigator and presented as a shared care model, which involves the General Practitioner (GP), a Nurse Navigator (NN) and the oncologist. AMA-AC was found to be feasible, greatly appreciated by patients and remarkably efficient for detecting complications during lymphoma survivorship [6]. However, this first study was essentially aimed at presenting the reliability of the AMA-AC program and therefore only dealt with a limited number of patients (*n* = 100) and a short follow-up (12 months).

In the current study, we prospectively recorded treatment-related complications, psychological disorders, return to work, life style and HRQoL in a cohort of 204 disease-free lymphoma survivors monitored for a minimum of 24 months, as set out in the AMA-AC program.

The current study aims to assess the proportion of patients with a significantly reduced quality of life after 2 years of post-cancer follow-up and to identify associated risk factors with a specific focus on physical events (such as infections, cardiovascular complications, neuropathy) as well as psychological disturbances occurring during this time period.

## Methods

Eligibility criteria were as follows: advanced Hodgkin’s lymphoma treated with a first line therapy consisting of a minimum of 6 cycles of ABVD or BEACOPP programs, Non-Hodgkin Lymphomas (NHL; B or T cell derived) treated with a minimum of 6 cycles of RCHOP, CHOP or equivalent (RACVBP, R-COPADEM, CHVP), with or without Autologous Stem Cell Transplantation (ASCT), according to the French intergroup LYSA recommendations. All patients were treated in the Hematology Department of the Toulouse University Medical Centre. Biopsy specimens were reviewed in our pathology department according to the Lymphopath procedure [15].

Patients who achieved complete response after therapy, as assessed by the Cheson criteria (including systematic PET at the end of therapy) [16] were asked to attend an initial AMA-AC consultation during which the oncologist and a NN described the program to the patients and their caregivers. Patients, as well as the GP performing clinical follow-up in ambulatory medicine, provided their written informed consent to participate in this study. This study was approved by the ethics committee of the Toulouse University Hospital (N°: 37–0712).

The AMA-AC survey has been described elsewhere [6]. Briefly, the patient visits his/her GP every 3 months during the first year, then every 6 months for the 4 following years for a total follow-up period of 5 years. GP appointments focus on detecting/recording physical events: symptoms compatible with relapse, drug-related sequela (neuropathy, arthralgia, osteoporosis, sexual and fertility problems), infections, non-hematological complications such as cardiovascular events or second cancers. All GPs complete a 41-item Clinical Report Form (CRF), which is sent by email to the NN.

Thereafter, the NN phones the patient at home to complete any outstanding CRF issues and to help the patient fill out the questionnaires dealing with psychological and social status. The, HRQoL is the main assessment criteria. HRQoL is evaluated using the self-reported French version of the SF-36 scale [17], administered at M0, M12 and M24 (M0 refers to the initiation of AMA-AC. M12 and M24 to the 12 and 24 months into AMA-AC, respectively). The 36 items of this list are divided into two subscales: The Physical Component Score (PCS) and the Mental Component Score (MCS), each is scored from 0 to 100 (excellent) with scores ≤50 equated with severe degradations in HRQoL.

Anxiety and depression were scored with the HAD scale [18]. As in our previous study, a score above 8 for either anxiety or depression was considered significant. PTSD (full or partial) were scored with the PCL (PTSD check-list) [19], with scores above 44 considered significant. Return to work or cohabitation status (living together or living alone) were also recorded. Finally, the NN also recorded Body Mass Index (BMI) and tobacco use. Physical and psychological events were qualified both as incidence and prevalence (incidence is the number of new patients affected over a period of time; prevalence is the number of patients affected at a given time).

The physical, psychological and social component records were subsequently sent to the oncologist who consolidated the three files and, where required, also intervened via the GP or directly interacted with the patient. In the AMA-AC program patients were not systematically asked to attend visits at the hospital; however, they were examined by the oncologist on request (within 1 week of issuing the request). The AMA-AC program was very well perceived, with only one patient refusing to sign the informed consent form and none of the GPs refusing.

Between January 2012 and May 2018, a total of 360 patients were prospectively included into the AMA-AC program. Among these, 258 patients were followed over M0-M24. This group consisted of 204 patients’ disease free at M24 and constituted the major interest group of the current study. Among the 54 remaining patients, we noted: 31 relapses (12.0%), 7 deaths (2.7%), 10 premature discontinuations of the AMA-AC surveys (3.8%) and 6 patients who moved to another region (2.3%). These patients were excluded from the analysis.

Clinical characteristics included individual parameters at diagnosis (gender, age, Charlson comorbidity index/CCI, ECOG performance status, health assurance coverage, level of education, cohabitation status, occupation and income), disease-related parameters (histology, Ann Harbor stage), and treatment-related parameters (conventional versus intensified, the latter being BEACOPP, RACVBP and intensification with front-line ASCT).

Data collection and analysis: an anonymized database was used to collect all information related to the cohort. This database was secured and managed by an external service device in accordance with recommendations of the appropriate regulatory committees. Quantitative variables of baseline patient characteristics were defined as means with standard deviations and categorical variables as percentages. We implemented a multivariate logistic regression model adjusted for variables statistically associated with outcome in the bivariate analyses, with an alpha risk of 20%. Interactions between the covariates were verified for each model. A two-sided *p*-value < 0.05 was considered as statistically significant for the multivariate model. Analysis between favorable and unfavorable groups for PCS and MCS were performed using the Chi2test with the Yates correction if relevant. Statistical analyses were performed using SAS® software version 9.4 (SAS institute, Cary, NC).

## Results

### Initial characteristics of patients

Information relative to socio-demographics, disease-related profile and type of treatment was collected at diagnosis. Results for the 204 patients are depicted in Table 1. Median age was 59 years (19–85). Only 25% of patients lived alone. Diffuse large B cell (DLBCL) was the most frequent histological subtype. The most common treatment was RCHOP (57%) followed by RACVBP (a French dose-dense RCHOP-derived regimen) (18.6%), ABVD (9%) and BEACOPP (6.9%). A total of 52 patients were treated with intensive treatments (RACVBP, BEACOPP, ASCT). At diagnosis, 23% of patients used tobacco and 8.8% of patients had a BMI > 30.

**Table 1:** Characteristics of the 204 patients included in the AMA-AC program

Patient characteristics at the entry to AMA-AC: *n* = number of cases documented	(*n* = 204)
Gender Men (*n* = 204)	113 (55.4%)
Age (years)	
Mean ± sd	55.2 ± 15.4
Median (Min; Max)	59 (19–85)
Health insurance (*n* = 204)	
General health system	175 (85.8%)
Others (Agriculture, freelancers)	29 (14.2%)
Level of education (*n* = 205)	
Lower educational status (≤high school degree)	116 (56.9%)
Higher educational status (>high school degree)	88 (43.1%)
Disease-related characteristics	
Histology (*n* = 204)	
Diffuse large B-cell lymphoma (DLBCL)	129 (63.2%)
Hodgkin lymphoma and other NHLs	75 (36.8%))
Ann Arbor stage (*n* = 203)	
I/ II	45 (22.2%)
III/ IV	158 (77.8%)
Performance status (*n* = 204)	
≤ 1	181 (88.7%)
≥ 2	23 (11.3%)
Charlson comorbidity index (*n* = 204)	
0	54 (26.5%)
1	35 (17.1%)
≥ 2	115 (56.4%)
Type of treatment line (*n* = 204)^a^	
Conventional	152 (74.5%)
Intensified	52 (25.5%)
Cohabitation status (*n* = 196)	
Living together (married, living in partnership)	147 (75.0%)
Living alone (single, divorced, widowed)	49 (25.0%)
Occupational status (*n* = 203)	
Active (employed)	110 (54.2%)
Not active (without employment, retired, jobless)	93 (45.8%))
Income / month (*n* = 204)	
No salary	8 (3.9%)
< 380€ - 1070€	29 (14.2%)
> 1070€ - 1830€	70 (34.3%)
> 1830€ - 2290€	18 (8.8%)
> 2290€ - ≥4570€	48 (23.6%)
No comment	31 (15.2%)

^a^Type of treatment line: Conventional: see text

### Physical events: (Table 2)

Infections (all grades together) were the most frequent complications. Approximately 50–70 infectious events occurred during the different segments of the M0-M24 period; predominantly bronchitis and sinusitis during the first year, while urinary and genital infections were more frequent during the second year (data not shown). Most of patients presented with multiple infections across their trajectories. This explains the high cumulative number of events. No patient died from infection. Although prophylaxis against pulmonary pneumocystosis with sulfamethoxazole/trimethoprime (systematically given during chemotherapy) was stopped at M0, no patient developed pulmonary pneumocystosis. Despite infections being benign in the vast majority of cases, they did entail GP visits, the administration of antibiotics and caused fatigue. Less frequent, but disabling, were neuropathy and arthralgia (all grades together). However, as shown in Table 2, the spectrum of drug-related toxicity slightly shifted with a decrease in prevalence of arthralgia and peripheral neuropathy over time, with peripheral neuropathy still affecting some 8% of patients at M24.

**Table 2:** Prevalence of complications at different time periods for the entire cohort (*n* = 204 patients)

complications	M0-M3 n (%)	M3-M6 n (%)	M6-M9 n (%)	M9-M12 n (%)	M12-M18 n (%)	M18-M24 n (%)
Infections	51 (25.1)	76 (37.6)	77 (38.3)	71 (35.5)	50 (25.0)	52 (26.1)
Arthralgia	78 (38.2)	80 (39.4)	66 (32.8)	70 (35.0)	53 (26.5)	49 (24.6)
Peripheral neuropathy	50 (24.5)	40 (19.7)	36 (17.9)	32 (16.0)	22 (11.0)	16 (8.0)
Gastritis	20 (9.8)	22 (10.8)	21 (10.4)	14 (7.0)	9 (4.5)	9 (4.5)
Erectile dysfunction	26 (12.7)	26 (12.8)	29 (14.4)	23 (11.5)	23 (11.5)	23 (11.5)
Libido disturbances	26 (12.7)	18 (8.9)	20 (10.0)	15 (7.5)	16 (8.0)	20 (10.0)
Osteoporosis	8 (3.9)	9 (4.4)	8 (4.0)	11 (5.5)	15 (7.5)	11 (5.5)
Hypogammaglobulinemia	50 (24.4)	ND	ND	68 (33.1)	ND	49 (23.9)
Cardio-vascular events	14 (6.8)	9 (4.4)	11 (5.3)	17 (8.2)	16 (7.8)	11 (5.5)
Thyroid (benign)	3 (1.4)	3 (1.4)	3 (1.4)	4 (1.9)	3 (1.4)	3 (1.4)
Urogenital^a^	14 (6.8)	12 (5.8)	15 (7.5)	11 (5.5)	15 (7.5)	19 (9.2)
Ear, nose and throat^a^	15 (7.5)	8 (3.9)	15 (7.5)	15 (7.5)	8 (3.9)	9 (4.5)
Pulmonary^a^	19 (9.2)	14 (7.0)	11 (5.3)	17 (8.2)	9 (4.5)	16 (7.8)
Second cancers	3 (1.4)	4 (1.9)	2 (0.9)	3 (1.4)	5 (2.4)	6 (2.9)

^a^ cancers and infections were excluded

Gammaglobulin (Ig) concentration was measured at M0, M12 and M24. Hypogammaglobulinemia (Ig concentration < 8 g/L) was common at M0 (24.4%). At M24, approximately one third of patients were still affected (all levels together). This group of patients were heterogeneous comprising not only patients with mild hypogammaglobulinemia (between 8 g/L and 3 g/L), most often asymptomatic and rarely treated with Ig prophylaxis, but also patients with severe hypogammaglobulinema (< 3 g/L), often infected, who received Ig prophylaxis early in their trajectory, resulting in the normalization of Ig levels. Altogether, it appeared that hypogammaglobulinemia was a relatively common complication but it was nevertheless much less frequent than infections.

Sexuality was disrupted with decreased libido in men and in women as well as erectile dysfunction in men (23% at M24) with a strong demand for phosphodiesterase inhibitors. 10% of patients used oral contraceptives. One patient became pregnant during the course of the study.

The prevalence of cardiovascular complications was relatively stable over the different time-periods, with approximately 27 events during the M12-M24 period. Over the entire cohort and the M0-M24 trajectory, we noted acute myocardial infarctions (*n* = 1), cardiac insufficiencies (*n* = 11), coronaropathies (*n* = 10), arrhythmias (*n* = 38), phlebitis (*n* = 2), arteritis (*n* = 19) for a total of 81 events (one patient may display more than one event), this was unexpectedly high, based on our previous report. Cardiovascular events were therefore the second most frequently observed event after infections among lymphoma survivors during the first 2 years of AMA-AC follow-up.

Second cancers were also major concerns with 23 cases observed over the 2-year period (11.2%): skin cancer: 5, prostate cancer: 4, thyroid cancer: 4, lung cancer: 3, breast cancer: 2, pancreatic cancer: 2, stomach cancer: 1, colon cancer: 1 and leukemia: 1.

### HRQoL

**Fig. 1:**
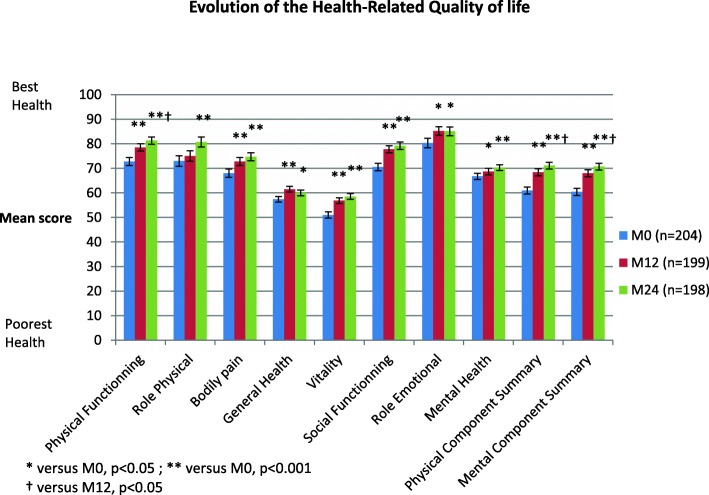
Health-related quality of life (SF-36) evaluation with the SF-36 at entry into the AMA-AC program (*n* = 204 patients), after 12 months (*n* = 199 patients) and after 24 months (*n* = 198 patients)

**Fig. 2:**
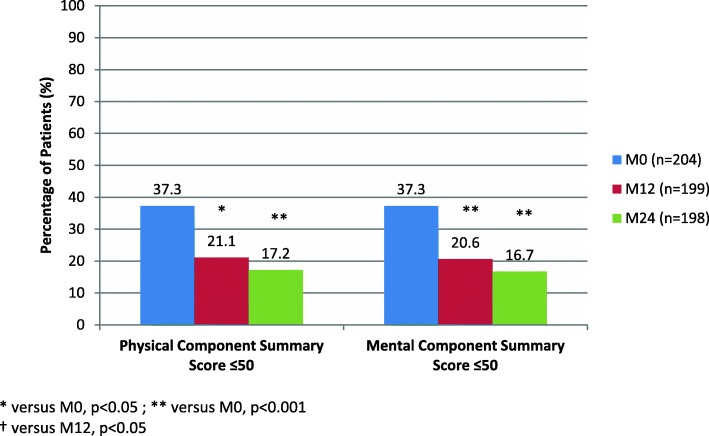
Evolution of patients with altered health-related quality of life (scores ≤50)

For the entire cohort, HRQoL progressively increased from M0 to M24 (Fig. 1). All components were significantly improved between M0 and M24 with the exception of general and mental health. However, the current study identified a group of patients at M24 for whom HRQoL remained poor (PCSs or MCSs < 50 according to SF-36). In our previous report, we observed that one fifth of patients displayed HRQoL scores < 50 at M12 [6]. Whether these patients’ HRQoL scores improved during the next period of time could however not be determined. In the current study, based on a 2-year follow up and with twice the number of patients, these patients now represent 21.1 and 20.6% for PCS and MCS, respectively at M12 and as high as 17.2 and 16.7% for PCS and MCS, respectively at M24 (the differences between M12 and M24 were not significant) (Fig. 2).

The current study therefore confirms the presence of a group of HL and NHL patients with complete responses and potentially cured of their disease, who still displayed a persistent and profound alteration in HRQoL. Because this is a novel finding which has not been previously reported in the literature, we further investigated specific factors associated with these patient profiles.

### Factors associated with low PCS scores

Univariate analysis indicated that some initial characteristics such as advanced age, low educational level and being unemployed were associated with PCS scores of ≤50 at M24 (Table 3). Moreover, other events which occurred during the first 12 months were also associated with decreased PCS scores: MCS status at M12, occurrence or persistence of PTSD, depression, cardiovascular events and neuropathy. Interestingly, cohabitation status, financial resources, comorbidity and infections as well as most parameters related to the disease (histology, stage) or to the treatment itself (conventional versus intensified) were not associated with low PCS. However, multivariate analysis showed that only the occurrence of PTSD was highly predictive of an altered HRQoL (Table 4).

**Table 3:** Comparison between groups with favorable (PCS scores > 50) and unfavorable (PCS scores ≤50) HRQoL at M24

	Favorable PCS (scores > 50) *n* = 164	Unfavorable PCS (scores ≤50) *n* = 34	OR CI 95%	*P*-Value Univariate
Gender				
N	164	34		
Missing	0	0		
Men	90 (84.9%)	16 (15.1%)	0.731 [0.349,1.532]	0.4065
Women (Réf.)	74 (80.4%)	18 (19.6%)		
Age (years) at AMA-AC entry				
N	164	34		
Missing	0	0	Unit = 10	
Mean (s.d.)	53.8 (15.7)	62.4 (12.0)	1.531 [1.144,2.050]	0.0042
Level of education				
N	164	34		
Missing	0	0		
Higher education status (> high school degree) (Réf.)	77 (89.5%)	9 (10.5%)		
Lower education status (≤ high school degree)	87 (77.7%)	25 (22.3%)	2.458 [1.081,5.589]	0.0318
Cohabitation status				
N	159	30		
Missing	5	4		
Living alone (single, divorced, widowed) (Réf.)	43 (89.6%)	5 (10.4%)		
Living together (married, living in partnership)	116 (82.3%)	25 (17.7%)	1.853 [0.667,5.150]	0.2367
Occupational status				
N	159	30		
Missing	5	4		
Active (employed) (Réf.)	87 (89.7%)	10 (10.3%)		
Not active (unemployed, retired, jobless)	72 (78.3%)	20 (21.7%)	2.417 [1.064,5.491]	0.0351
Geographical area				
N	159	30		
Missing	5	4		
Rural (Réf.)	76 (86.4%)	12 (13.6%)		
Urban/Semi-urban	83 (82.2%)	18 (17.8%)	1.373 [0.621,3.038]	0.4334
Histology				
N	164	34		
Missing	0	0		
Diffuse large B-cell lymphoma (DLBCL) (Réf.)	104 (82.5%)	22 (17.5%)		
Others (FL, HL, MCL)	60 (83.3%)	12 (16.7%)	0.945 [0.437,2.046]	0.8867
Stage in class				
N	163	34		
Missing	1	0		
I / II (Réf.)	36 (81.8%)	8 (18.2%)		
III / IV	127 (83.0%)	26 (17.0%)	0.921 [0.384,2.209]	0.8542
ECOG in class				
N	164	34		
Missing	0	0		
≤ 1 (Réf.)	146 (83.4%)	29 (16.6%)		
≥ 2	18 (78.3%)	5 (21.7%)	1.399 [0.481,4.069]	0.5379
CHARLSON SCORE (class)				
N	113	31		
Missing	51	3		
1 (Réf.)	27 (77.1%)	8 (22.9%)		
≥ 2	86 (78.9%)	23 (21.1%)	0.903 [0.362,2.250]	0.8260
Type of treatment				
N	164	34		
Missing	0	0		
Conventional	112 (81.2%)	26 (18.8%)	1.508 [0.640,3.557]	0.3476
Intensified (Réf.)	52 (86.7%)	8 (13.3%)		
Calculated BMI (kg/m^2^)				
N	158	30		
Missing	6	4		
Mean (s.d.)	24.1 (4.0)	24.7 (5.1)	1.033 [0.945,1.130]	0.4730
Physical Components Summary scale (class) at M12				
N	164	33		
Missing	0	1		
Unfavorable (≤50)	21 (51.2%)	20 (48.8%)	10.476 [4.545,24.147]	<.0001
Favorable (> 50) (Réf.)	143 (91.7%)	13 (8.3%)		
Mental Components Summary scale (class) at M12				
N	164	33		
Missing	0	1		
Unfavorable (≤50)	23 (57.5%)	17 (42.5%)	6.514 [2.890,14.680]	<.0001
Favorable (> 50) (Réf.)	141 (89.8%)	16 (10.2%)		
At least one occurrence of depression between 3 and 18 months (HAD depression scale > 8)				
N	163	34		
Missing	1	0		
No (Réf.)	125 (86.8%)	19 (13.2%)		
Yes	38 (71.7%)	15 (28.3%)	2.597 [1.205,5.599]	0.0149
At least one occurrence of anxiety between 3 and 18 months (HAD anxiety scale > 8)				
N	164	34		
Missing	0	0		
No (Réf.)	101 (84.2%)	19 (15.8%)		
Yes	63 (80.8%)	15 (19.2%)	1.266 [0.600,2.670]	0.5362
At least one occurrence of PTSD between 3 and 18 months				
N	161	32		
Missing	3	2		
No (Réf.)	140 (89.2%)	17 (10.8%)		
Yes	21 (58.3%)	15 (41.7%)	5.882 [2.560,13.519]	<.0001
At least one occurrence of cardiovascular disorders between 3 and 18 months				
N	163	34		
Missing	1	0		
No (Réf.)	143 (86.1%)	23 (13.9%)		
Yes	20 (64.5%)	11 (35.5%)	3.420 [1.451,8.060]	0.0049
At least one infection occurring between 3 and 18 months				
N	163	34		
Missing	1	0		
No (Réf.)	40 (81.6%)	9 (18.4%)		
Yes	123 (83.1%)	25 (16.9%)	0.903 [0.389,2.095]	0.8128
At least one occurrence of arthralgia between 3 and 18 months				
N	164	34		
Missing	0	0		
No (Réf.)	54 (90.0%)	6 (10.0%)		
Yes	110 (79.7%)	28 (20.3%)	2.291 [0.895,5.864]	0.0839
At least one occurrence of neuropathy between 3 and 18 months				
N	164	34		
Missing	0	0		
No (Réf.)	110 (87.3%)	16 (12.7%)		
Yes	54 (75.0%)	18 (25.0%)	2.292 [1.085,4.842]	0.0298

**Table 4:** Multivariate analysis for unfavorable PCS scores of ≤50 at M24 HRQoL (*N* = 192 patients)

Odds Ratio Estimates and Wald Confidence Intervals			
Odds Ratio	Estimate	95% Confidence Limits	
PCS CLASS at M12: Unfavorable (≤50) vs Favorable (> 50)	5.333	1.974	14.409
PTSD: Yes vs No	3.394	1.161	9.926
AGE: units = 10	1.569	1.090	2.257

Multivariate model with the parameters retained after the univariate analyzes (cf. previous table with *p*-value ≤0.10; value in bold in Table 3). The « Occupational status » variable with missing data was not included in the multivariate model. Given the missing data for all the parameters selected, the number of patients taken into account is 192

### Factors associated with low MCS scores

Univariate analysis showed that a low educational level, histology (DLBCL), PCS status at M12 and the occurrence of infections, cardiovascular events, depression and PTSD were associated with MCS scores of ≤50 (Table 5). Moreover, multivariate analysis showed that only PCS scores and histology (DLBCL versus HL, MCL, FL), but not social-determinants, were independent risk factors for persistent and severe degradation of mental HRQoL components (Table 6).

**Table 5:** Comparison between groups with favorable MCS (scores > 50) and unfavorable MCS (scores ≤ 50) HRQoL at M24

	Favorable MCS (scores > 50) *n* = 165	Unfavorable MCS (scores ≤50) *n* = 33	OR CI 95%	*P*-Value Univariate
Gender				
N	165	33		
Missing	0	0		
Men	86 (81.1%)	20 (18.9%)	1.413 [0.659,3.028]	0.3738
Women (Réf.)	79 (85.9%)	13 (14.1%)		
Age (years) at AMA-AC entry				
N	165	33		
Missing	0	0		
Mean (s.d.)	54.9 (15.8)	57.3 (13.9)	1.111 [0.865,1.427]	0.4095
Level of education				
N	165	33		
Missing	0	0		
Higher education status (> high school degree) (Réf.)	77 (89.5%)	9 (10.5%)		
Lower education status (<= high school degree)	88 (78.6%)	24 (21.4%)	2.333 [1.023,5.324]	0.0441
Cohabitation status				
N	161	28		
Missing	4	5		
Living alone (single, divorced, widowed) (Réf.)	42 (87.5%)	6 (12.5%)		
Living together (married, living in partnership)	119 (84.4%)	22 (15.6%)	1.294 [0.491,3.409]	0.6020
Occupational status				
N	161	28		
Missing	4	5		
Active (employed) (Réf.)	82 (84.5%)	15 (15.5%)		
Not active (unemployed, retired, jobless)	79 (85.9%)	13 (14.1%)	0.900 [0.402,2.011]	0.7965
Geographical area				
N	161	28		
Missing	4	5		
Rural (Réf.)	76 (86.4%)	12 (13.6%)		
Urban/Semi-urban	85 (84.2%)	16 (15.8%)	1.192 [0.530,2.680]	0.6706
Histology				
N	165	33		
Missing	0	0		
Diffuse large B-cell lymphoma (DLBCL) (Réf.)	110 (87.3%)	16 (12.7%)		
Others (FL, HL, MCL)	55 (76.4%)	17 (23.6%)	2.125 [0.998,4.524]	0.0505
Stage in class				
N	164	33		
Missing	1	0		
I / II (Réf.)	36 (81.8%)	8 (18.2%)		
III / IV	128 (83.7%)	25 (16.3%)	0.879 [0.365,2.114]	0.7732
ECOG in class				
N	165	33		
Missing	0	0		
≤ 1 (Réf.)	148 (84.6%)	27 (15.4%)		
≥ 2	17 (73.9%)	6 (26.1%)	1.935 [0.700,5.349]	0.2035
CHARLSON SCORE (class)				
N	117	27		
Missing	48	6		
1 (Réf.)	25 (71.4%)	10 (28.6%)		
> =2	92 (84.4%)	17 (15.6%)	0.462 [0.188,1.133]	0.0917
Type of treatment				
N	165	33		
Missing	0	0		
Conventional	115 (83.3%)	23 (16.7%)	1.000 [0.443,2.255]	1.0000
Intensified (Réf.)	50 (83.3%)	10 (16.7%)		
Calculated BMI (kg/m^2^)				
N	160	28		
Missing	5	5		
Mean (s.d.)	24.1 (4.0)	24.6 (4.9)	1.023 [0.932,1.123]	0.6298
Physical Components Summary scale (class) at M12				
N	165	32		
Missing	0	1		
Unfavorable (≤50)	19 (46.3%)	22 (53.7%)	16.905 [6.961,41.054]	<.0001
Favorable (> 50) (Réf.)	146 (93.6%)	10 (6.4%)		
Mental Components Summary scale (class) at M12				
N	165	32		
Missing	0	1		
Unfavorable (≤50)	19 (47.5%)	21 (52.5%)	14.670 [6.133,35.091]	<.0001
Favorable (> 50) (Réf.)	146 (93.0%)	11 (7.0%)		
At least one occurrence of depression between 3 and 18 months (HAD depression scores > 8)				
N	164	33		
Missing	1	0		
No (Réf.)	128 (88.9%)	16 (11.1%)		
Yes	36 (67.9%)	17 (32.1%)	3.778 [1.738,8.211]	0.0008
At least one occurrence of anxiety between 3 and 18 months (HAD anxiety scale > 8)				
N	165	33		
Missing	0	0		
No (Réf.)	107 (89.2%)	13 (10.8%)		
Yesi	58 (74.4%)	20 (25.6%)	2.838 [1.317,6.117]	0.0078
At least one occurrence of anxiety between 3 and 18 months (HAD-anxiety scale > 8)				
N	165	33		
Missing	0	0		
No (Réf.)	107 (89.2%)	13 (10.8%)		
Yesi	58 (74.4%)	20 (25.6%)	2.838 [1.317,6.117]	0.0078
At least one occurrence of PTSD between 3 and 18 months				
N	161	32		
Missing	4	1		
No (Réf.)	142 (90.4%)	15 (9.6%)		
Yes	19 (52.8%)	17 (47.2%)	8.471 [3.645,19.688]	<.0001
At least one occurrence of cardiovascular disorders between 3 and 18 months				
N	164	33		
Missing	1	0		
No (Réf.)	142 (85.5%)	24 (14.5%)		
Yes	22 (71.0%)	9 (29.0%)	2.420 [0.996,5.882]	0.0511
At least one infection occurring between 3 and 18 months				
N	164	33		
Missing	1	0		
No (Réf.)	41 (83.7%)	8 (16.3%)		
Yes	123 (83.1%)	25 (16.9%)	1.041 [0.436,2.488]	0.9273
At least one occurrence of arthralgia between 3 and 18 months				
N	165	33		
Missing	0	0		
No (Réf.)	51 (85.0%)	9 (15.0%)		
Yes	114 (82.6%)	24 (17.4%)	1.193 [0.518,2.747]	0.6785
At least one occurrence of neuropathy between 3 and 18 months				
N	165	33		
Missing	0	0		
No (Réf.)	108 (85.7%)	18 (14.3%)		
Yes	57 (79.2%)	15 (20.8%)	1.579 [0.741,3.365]	0.2366

**Table 6:** Multivariate analysis for unfavorable* MCS scores of ≤50 at M24 HRQoL (*n* = 197 patients)

Odds Ratio Estimates and Wald Confidence Intervals			
Odds Ratio	Estimate	95% Confidence Limits	
Histology: Hodgkin lymphoma and other NHL vs Diffuse large B-cell lymphoma (DLBCL)	2.479	0.972	6.322
MCS CLASS at M12: Unfavorable (≤50) vs Favorable (> 50)	4.005	1.090	14.712
PCS CLASS at M12: Unfavorable (≤50) vs Favorable (> 50)	6.522	1.784	23.850

Multivariate model with the parameters retained after the univariate analyzes (cf. previous table with *p*-value ≤0.10, value in bold in Table 5). Given the missing data for all the parameters selected, the number of patients taken into account is 197

### Psychological disorders

**Fig. 3:**
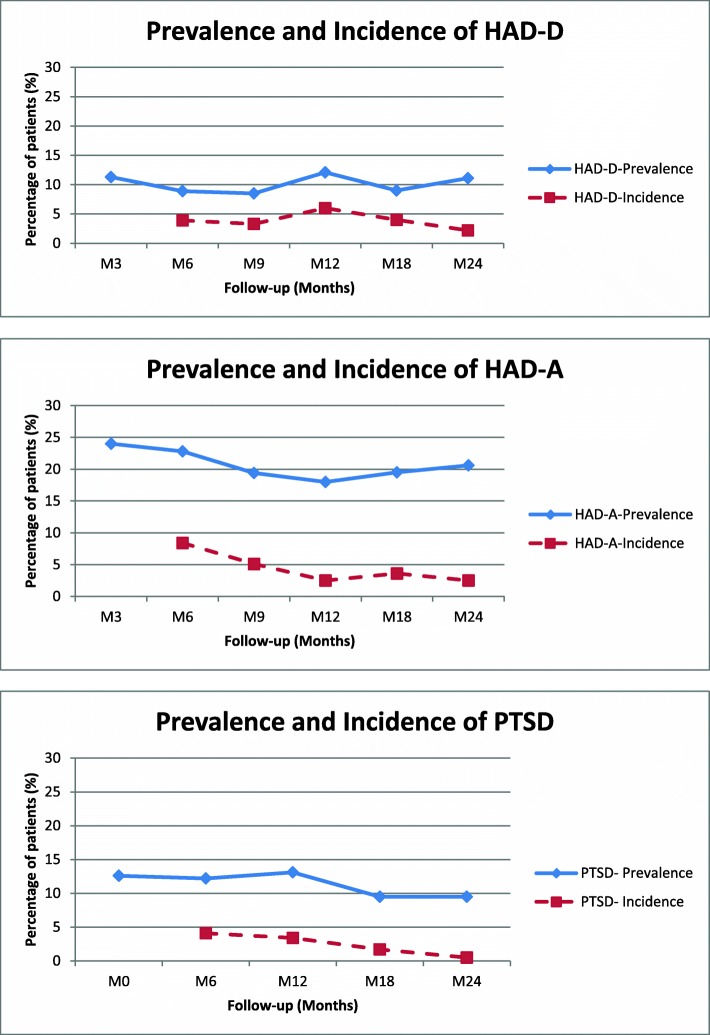
Incidence and prevalence of psychological disorders

The prevalence as well as the incidence of anxiety (HAD-A), depression (HAD-D) and PTSD are depicted in Fig. 3. Compared to our previous work [6], it should be noted that the incidence of anxiety greatly decreased between M12 and M24. However, the prevalence of anxiety was approximately 20%. In contrast, the incidence of depression remained low and its prevalence was approximately 10% (M24).

Similarly, the incidence of PTSD decreased over time and very few new cases of PTSD were observed beyond 12 months. The prevalence was nevertheless relatively high (approximately 10% of patients during the M0-M24 period).

### Relationship between low HRQoL scores (PCS < 50 and MCS ≤50) and occupational activity, tobacco use and being overweight during the survey

Occupational activity: out of 203 patients, 110 were employed when chemotherapy was started while 93 were retired and one was student. These patients were equally distributed in the two groups: 90/203 (44.3%) in the “favorable” group and 17/34 (50%) in the “unfavorable group”. During therapy, only 23.5% continued to work. At M24, 77/110 patients (70%) worked (mostly full time), 15.4% of patients were still on sick leave (temporary discontinuation of occupational activity) and only 11.8% received disability payments and permanently discontinued their occupation. However, we observed striking differences between the favorable and unfavorable groups. Indeed, the favorable group with HRQoL scores > 50 (PCS or MCS) had high rates of occupational activity and were less likely to not be in permanent employment or to have ceased transitionary employment. Compared to the unfavorable group, these differences were highly significant (Table 7).

**Table 7:** Relationship between low HRQoL scores (PCS ≤50 or MCS ≤50) and occupational activity, tobacco use and being overweight during the survey

a Low Physical HRQoL scores (PCS < 50)			
	favorable group (PCS scores > 50 at M24) *n* = 164	unfavorable group (PCS scores ≤ 50 at M24) *n* = 34	*P* value
Patients with occupational activity (110 employed at diagnosis)	number of patients who met the criteria/ total number of patients	number of patients who met the criteria/ total number of patients	
M0	44/164 (26.8%)	6/34 (17.6%)	n.s.
M12	63/164 (38.4%)	6/34 (17.6%)	*p* < 0.05
M24	71/164 (43.3%)	6/34 (17.6%)	*p* < 0.01
Tobacco use	number of patients who met the criteria/total number of patients	number of patients who met the criteria/ total number of patients	
At diagnosis	33/164 (20.1%)	14/34 (41.2%)	*p* < 0.05
M0	9/164 (5.5%)	5/34 (14.7%)	n.s.
M12	9/164 (5.5%)	5/34 (14.7%)	n.s.
M24	9/164 (5.5%)	5/34 (14.7%)	n.s.
BMI > 30	number of patients who met the criteria/ total number of patients	number of patients who met the criteria/ total number of patients	
M0	18/164 (11.0%)	7/34 (20.6%)	n.s
M12	19/164 (11.6%)	6/34 (17.6%)	n.s
M24	24/164 (14.6%)	7/34 (20.6%)	n.s
b Low Mental HRQoL scores (MCS < 50)			
	favorable group (MCS scores > 50 at M24) *n* = 165	unfavorable group (MCS scores ≤ 50 at M24) *n* = 33	*P* value
Patients with occupational activity (110 employed at diagnosis)	number of patients who met the criteria/ total number of patients	number of patients who met the criteria/ total number of patients	
M0	44/165 (26.7%)	6/33 (18.2%)	n.s.
M12	63/165 (38.2%)	6/33 (18.2%)	*p* < 0.05
M24	71/165 (43.0%)	6/33 (18.2%)	*p* < 0.05
Tobacco use			
Diagnosis	33/165 (20.0%)	14/33 (42.4%)	*p* < 0.05
M0	9/165 (5.5%)	5/33 (15.2%)	n.s.
M12	9/165 (5.5%)	5/33 (15.2%)	n.s.
M24	9/165 (5.5%)	5/33 (15.2%)	n.s.
BMI > 30			
M0	18/165 (10.9%)	7/33 (21.2%)	n.s.
M12	19/165 (11.5%)	6/33 (18.2%)	n.s.
M24	24/165 (14.5%)	7/33 (21.2%)	n.s.

Weight gain: although the percentage of obese patients (BMI > 30 Kg/m^2^) increased during chemotherapy, their weight remained stable during the post-treatment period. No difference was observed between favorable and unfavorable groups (Table 7).

Tobacco use: tobacco use was very uncommon at M24 since users represented only 7% of the total (14/198). This percentage was higher in the unfavorable group versus the favorable group but the difference was not significant. It is important to note that the incidence of tobacco use at M0 (just after chemotherapy) was three times lower compared to tobacco use at diagnosis (6.8% versus 23%) (Table 7). Intriguingly, tobacco use at diagnosis was higher in the unfavorable group (41.1%) compared to the favorable one (19.4%) (*p* = 0.011).

## Discussion

The aim of this prospective cohort study was to investigate the sequence of medical, psychological and social events in patients successfully treated for lymphoma and followed-up during a two-year period in the AMA-AC program. This study indicates that more than half of patients were substantially impacted by disabling events, with infections, neuropathy, psychological disorders and limitations to return to work encountered most frequently. Most of these complications occurred during the first year of follow-up, with their incidence dramatically reduced during the second year. In the majority of cases, patients’ HRQoL recovered over time from the end of chemotherapy to the beginning of the third year of follow-up. The current study nevertheless identified that approximately 20% of patients displayed profound and sustained HRQoL alterations. These patients indeed presented with specific and yet previously undescribed risk factors.

We have previously described the principles, modality and feasibility of the AMA-AC program, a shared care model involving GP, an oncologist and a nurse managing the patient-navigator derived program [6]. A total of 360 patients were enrolled into the AMA-AC program from January 2012 to May 2018 (with 204 of these included in the current study). Our second AMA-AC study also confirmed some features previously reported in the pilot study. AMA-AC was well-accepted by the vast majority of patients and GPs, although the procedure was somewhat time-consuming for GPs (15–20 min). In the latter part of the follow-up period, patients, caregivers and GPs overwhelmingly supported the procedure. Among the 360 patients followed, none missed the GP visit or the phone call from the NN. We observed that every patient completed and returned all questionnaires. AMA-AC was also very productive for the concertation between the oncologist and the other specialists (e.g. cardiologists) directly or through the NN. Perhaps more importantly, at the initial phase of lymphoma survivorship, AMA-AC provided a point of contact and an appropriate referral to support services which mitigated the sense of abandonment at a time when the interaction with the treating team virtually stops [20]. In this regard, we believe that the proactive nurse-led intervention was an important channel of information and played a central role for reassurance [21]. AMA-AC therefore appears to be particularly well-perceived among the different models of care available to cancer survivors [22].

Physical events occurred frequently during the first year of follow-up, with all grades of infections being the most frequent event observed. Half of patients presented with at least one infectious episode during their first year, with the prevalence of episodes decreasing over the course of the second year. The vast majority of infections were grade 1 to 2 according to the National Cancer Institute Common Terminology Criteria for Adverse Events. Hospitalizations were uncommon and no fatal infections were observed. Infections occurring in the context of lymphoma survivorship are not well documented. However, in the French REMARC study, which compared lenalidomide maintenance and placebo in responding elderly patients with DLBCL treated with first-line RCHOP, Thieblemont et al. reported an infection rate as low as 6% in the control arm (*n* = 323 patients) [23]. The latter study however, only recorded grade 3 and grade 4 events. More generally, we believe that mild-to-moderate infections have been underestimated in lymphoma survivors, but that their recurrence makes infections an important contributor to discomfort, fatigue and absenteeism, as suggested by a qualitative study dealing with survivor experiences [24]. Importantly, infections were not systematically related to hypogammaglobulinemia, suggesting that immunosuppression occurred by other mechanisms.

Psychological disorders occurred frequently during the first year of follow-up (approximately 50% of patients were impacted by one or several disorders), with anxiety occurring most frequently. The incidence of anxiety decreased over time but the prevalence was approximately 20% over the 2-year period, a finding comparable to that reported in a large study of more than 50,000 cancer survivors [25] and similar to the incidence reported in lymphoma survivors [26]. Different factors have been shown to contribute to anxiety in cancer survivors, including unmet needs [27], fear of relapse as documented by we and others [28, 29] and fear of CT scan results [30].

In our study, depression was less common with incidence rates remaining below the 5% mark over the 2-year period. In the study described above [25], the prevalence of depression in cancer survivors (11.6%) was not significantly different to that of controls or spouses. However, other Hodgkin lymphoma studies have found that depression, associated or not with anxiety, may occur with time (beyond 5–7 years) at a relatively elevated rate (15–20%), especially among patients with additional risk factors such as incident mental disorders and/or low educational levels [31]. Such provocative findings still remain to be confirmed by other independent studies.

The prevalence of PTSD greatly varied according to diagnosis criteria. The PTSD Checklist–Civilian Version (PCL-C) was used to give an overall PLC-C score while sometimes evaluation referred to subscales scores for each diagnosis criteria such as re-experiencing, arousal or avoidance. For example, in a cross-sectional study dealing with 886 NHL long-term survivors (10.2 years), Smith and colleagues reported a mean PCL-C score of 27.0 with subscale scores of 6.9, 9.3, and 10.8 for re-experiencing, arousal and avoidance, respectively [9]. In this study, only 17% of patients displayed two of the three PTSD symptoms, defining “partial” or “full” PTSD. In accordance with prevailing studies, we have used the 44 threshold value to score PTSD although the term post-traumatic symptoms would perhaps be more appropriate. Using these criteria, we found that the prevalence of PTSD varied approximately 10–15% depending on the time of measurement. However, our study also showed that most of these post-traumatic events occurred during the initial phase of the trajectory, lasted over the 2-year period despite psychotherapy support. These observations were consistent with those of Smith and colleagues who described chronic forms of PTSD in NHL [32].

Decreases in HRQoL are of major concern during the post-cancer period of lymphoma patients. Indeed, the HRQoL is frequently reduced in lymphoma patients, a finding which is in contrast to the relatively favorable outcomes for lymphomas when compared with other cancers and sometimes even when compared to the more aggressive cancers [13]. Moreover, lymphoma patients, may experience a decrease in HRQoL for an extended period of time. In a cross-sectional study with long median follow-up (10 years) for example, Smith and colleagues reported that the altered HRQoL could last for years, largely beyond the initial 5 years, a period beyond which most clinical surveys have generally stopped [12]. Furthermore, standard surveys by oncologists may not be completely adapted to detect obstacles, as a significant fraction of survivors are not willing to talk about some sensitive components pertaining to HRQoL, such as social difficulties or sexual dysfunction. This specifically relates to women patients [33]. These observations suggest that HRQoL measurements over prolonged periods of time would be beneficial to detect and manage obstacles for facilitating a return to normality. In this respect, AMA-AC appeared to be a very simple procedure for monitoring HRQoL in routine practice, in cooperation with the patients themselves (in our experience, all patients were capable of filing in the SCF-36 form) and the NN.

Our findings were however not so pessimistic, at least not for the majority of patients. Indeed, although, general scores for physical and mental components of HRQoL were significantly affected immediately after therapy (M0), compared to controls, they gradually improved between M0 and M24 for most patients. The difference between M0 and M24 was significant for each score. However, in agreement with our previous study, we found that approximately one fifth of patients displayed poor (physical and/or mental scores ≤50) HRQoL at M12. Surprisingly, the HRQoL in this group of patients, did not recover during the entire 2-year follow-up period.

Several factors may contribute to alterations in HRQoL. As is the case in other cancer survivors, physical, psychological and social troubles converge to alter HRQoL. The rate and the magnitude of these complications are influenced by disease characteristics and treatment intensity. However, patient background may also profoundly affect physical and/or mental functional consequences or how these complications are perceived. This includes: advanced age (even if social impact may be greater in young patients) [34], comorbidity [35], socio-demographic disparities [36], personality traits [37], life style including tobacco use and weight gain [38]. Based on these considerations, we have compared most of these parameters between favorable (HRQoL > 50 at M24) and unfavorable groups (HRQoL ≤50 at M24). Altogether our results identified a risk profile associated with advanced age, low educational level, unemployment, and the occurrence of severe psychological disorders (more notably PTSD). In addition, a low score at M12 appeared to be predictive of an unfavorable outcome, suggesting that an HRQoL evaluation at the one-year mark may be important for detecting these types of patients.

Return to work, tobacco use and weight gain were also studied. It is interesting to note that tobacco use dramatically decreased from diagnosis to the end of therapy, likely due to recommendations given to patients during chemotherapy. However, weight gain was observed during the therapy period as previously described [38]. More generally, it appears that life style tends to be neglected by cancer survivors. Thus, a US cross-sectional study based on 566 NHL survivors showed that only 11% of patients met all 4 American Cancer Society health recommendations (physical activity, fruit and vegetable intake, body weight and tobacco use) [38]. Return to work was also evaluated. Compared to the favorable group, the unfavorable group displayed a very low rate of return to work and a high cessation of permanent employment at M24. In contrast, more than 75% of patients who had an occupation at diagnosis in the favorable group, continued or went back to work at a rate similar to, if not better than that described in the national French survey for cancer survivors [39].

Our study suffers from several limitations. Firstly, there was a selection bias illustrated by the relatively low median age, high performance status and low comorbidity. This is in agreement with our regional care organization which favors recruitment by the academic center of candidates for intensive therapy, older patients being preferentially treated by non-academic institutions. Thus, it is possible that the current study underestimates the rate and intensity of medical events as well as their psychosocial consequences. Secondly, we were missing some data points. For example, pertaining to life style, physical exercise and diet management were not evaluated, even though previous studies have documented these parameters as important contributors for preserving HRQoL [38]. Thirdly, some histological subtypes, such as HL, were underrepresented. Finally, other important components of HRQoL, such as fatigue which is major concern in HL [7] and NHL [8], were not been investigated.

## Conclusion

This prospective study provides evidence that lymphoma survivorship is punctuated by a number of physical and psychological events which have important functional consequences and all contribute to a reduction in HRQoL. Although physical and mental components improve over the first 2-year period, approximately 20% of patients display persistent HRQoL alterations associated with low rates of return to work. One of the most significant risk factors consists in elevated PCS or MCS scores at M12. Thus, a complete HRQoL evaluation at M12, as performed in the AMA-AC program, appears critical for detecting high-risk patients. This patient group would benefit from targeted interventions such as psychotherapy, social support and rehabilitation. From our study, we believe that GPs can take over from the oncologist after the 2-year mark in the case of lymphoma survivors who are well at M12 (80% of patients). In contrast, consultations with the oncologist are required all along the trajectory of risk patients, to provide personalized physical and/or psychosocial support, in addition to the care provided by the NN.

## Data Availability

Aggregate data are available on request from the corresponding author.

## Abbreviations

ABVD: Adriamycin, Bleomycin, vinblastine, Dacarbazine
AMA-AC: Ambulatory Medical Assistance for After-cancer
BEACOPP: Bleomycin, etoposide, Adriamycin, cyclophosphamide, Oncovin, Procarbazine, Prednisone
CHVP: Cyclophosphamide Adriamycin, VP16, Prednisone
CRF: Clinical Report Form
DLBCL: Diffuse Large B cell Lymhomas
GP: General Practitioner
HL: Hodgkin Lymphoma
HRQoL: Health-Related Quality of Life
MCS: Mental Component Score
NHL: Non-Hodgkin Lymphoma
NN: Nurse Navigator
PCS: Physical Component Score
PTSD: Post-Traumatic Stress Disorder
RACVBP: Rituximab, Adriamycin Cyclophosphamide, Vindesine, Bleomycin, Prednisone
RCHOP: Rituximab, Cyclophosphamide Adriamycin, Oncovin, Prednisone
